# Supplementary figures and images for: Vitrification of porcine immature oocytes and zygotes results in different levels of DNA damage which reflects developmental competence to the blastocyst stage
Source: PLoS One. 2023 Mar 17;18(3):e0282959. doi: 10.1371/journal.pone.0282959 (PMC10022796; doi:10.1371/journal.pone.0282959)

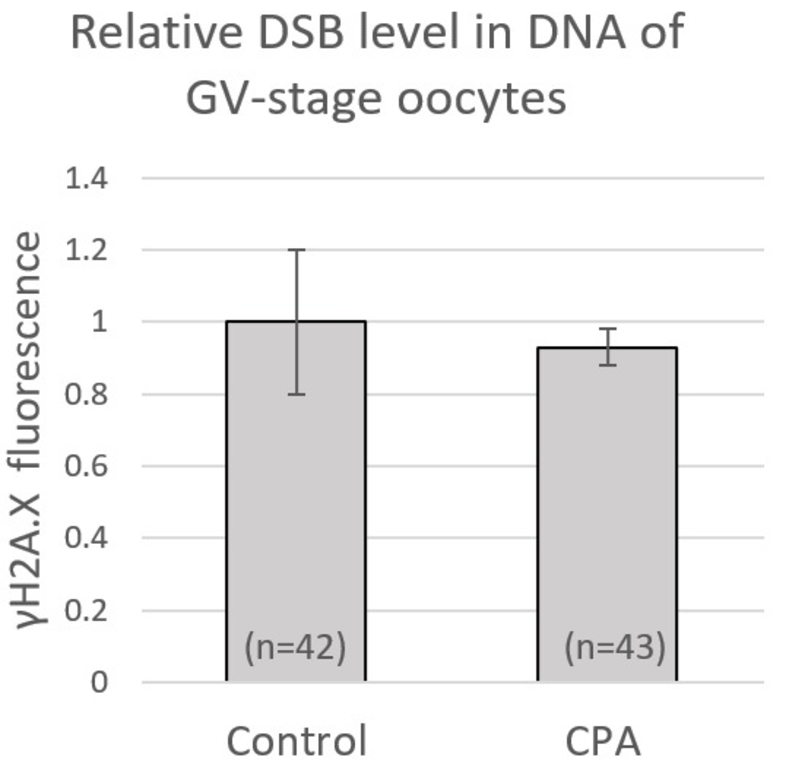

Supplement: S1 Fig — Data are shown as the mean ± SEM. A significant difference was not detected (P > 0.05) between the groups. The experiment was replicated three times. Total numbers of oocytes in each group are given in parentheses. (TIF) [file pone.0282959.s001.tif]

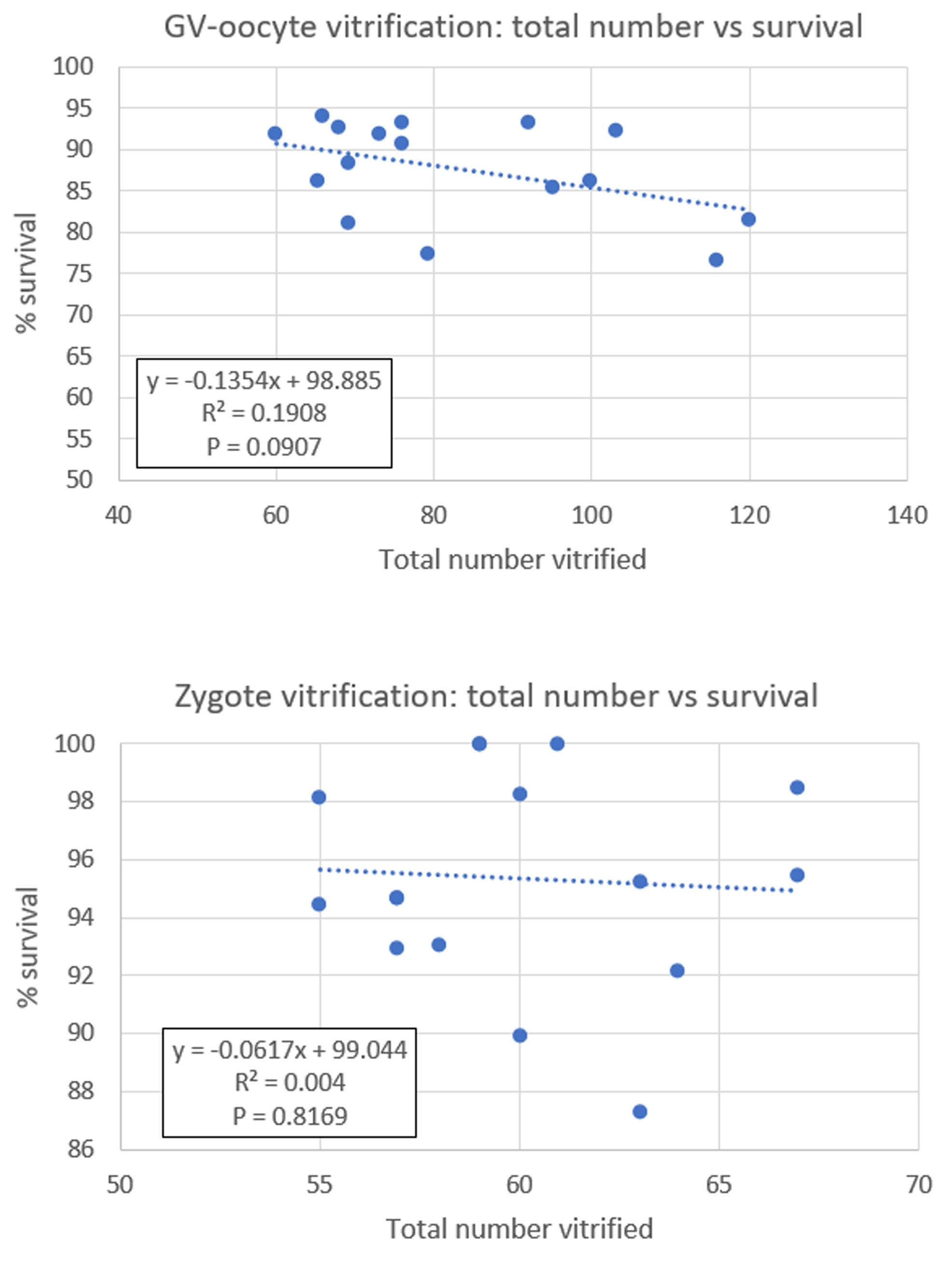

Supplement: S2 Fig — A significant correlation was not detected (P > 0.05). (TIF) [file pone.0282959.s002.tif]
