# Supplementary material for: Vitrification of porcine immature oocytes and zygotes results in different levels of DNA damage which reflects developmental competence to the blastocyst stage
Source: PLoS One. 2023 Mar 17;18(3):e0282959. doi: 10.1371/journal.pone.0282959 (PMC10022796; doi:10.1371/journal.pone.0282959)
Supplement: S1 Table — Six replications were performed. Percentage data are presented as mean ± SEM. No significant differences were detected among the treatment groups (P < 0.05).* Only live oocytes were used to assess fertilization status. **Characterized by one female pronucleus, one male pronucleus and 2 polar bodies. MPN = male pronucleus. (DOCX) [file pone.0282959.s003.docx]

| **S1 Table.** **Fertilization status 10 h after IVF of porcine oocytes vitrified either at the GV stage (GV-vitrified) or at the zygote stage (Zyg-vitrified)**. | | | | | |
| --- | --- | --- | --- | --- | --- |
| **Treatment**  **groups** | **No. of oocytes** | | | | |
|  | **Total*** | **Penetrated**  **(% total)** | **Normal****  **(% total)** | **MPN formation**  **(% penetrated)** | **Monospermy**  **(% penetrated)** |
| **Control** | 102 | 64 (62.3±4.3) | 20 (18.9±3.1) | 62 (97.0±1.8) | 28 (44.0±8.4) |
| **GV-vitrified** | 85 | 50 (56.8±6.8) | 14 (15.7±2.4) | 44 (88.3±7.3) | 25 (50.3±9.8) |
| **Zyg-vitrified** | 69 | 47 (62.6±10.6) | 12 (17.8±1.6) | 41 (83.0±7.6) | 18 (45.1±9.5) |
| Six replications were performed.  Percentage data are presented as mean ± SEM.  No significant differences were detected among the treatment groups (P < 0.05).  * Only live oocytes were used to assess fertilization status.  **Characterized by one female pronucleus, one male pronucleus and 2 polar bodies.  MPN = male pronucleus | | | | | |
